# Pluripotency factor binding and *Tsix *expression act synergistically to repress *Xist *in undifferentiated embryonic stem cells

**DOI:** 10.1186/1756-8935-4-17

**Published:** 2011-10-07

**Authors:** Tatyana B Nesterova, Claire E Senner, Janina Schneider, Tilly Alcayna-Stevens, Anna Tattermusch, Myriam Hemberger, Neil Brockdorff

**Affiliations:** 1 Developmental Epigenetics Group, Department of Biochemistry, University of Oxford, South Parks Road, Oxford, OX1 3QU, UK; 2 Epigenetics Programme, The Babraham Institute, Babraham Research Campus, Cambridge CB22 3AT, UK; 3 Molecular Haematology Unit, Weatherall Institute of Molecular Medicine, John Radcliffe Hospital, Oxford University, Oxford, OX3 9DS, UK

## Abstract

**Background:**

Expression of *Xist*, the master regulator of X chromosome inactivation, is extinguished in pluripotent cells, a process that has been linked to programmed X chromosome reactivation. The key pluripotency transcription factors Nanog, Oct4 and Sox2 are implicated in *Xist *gene extinction, at least in part through binding to an element located in *Xist *intron 1. Other pathways, notably repression by the antisense RNA *Tsix*, may also be involved.

**Results:**

Here we employ a transgene strategy to test the role of the intron 1 element and *Tsix *in repressing *Xist *in ES cells. We find that deletion of the intron 1 element causes a small increase in *Xist *expression and that simultaneous deletion of the antisense regulator *Tsix *enhances this effect.

**Conclusion:**

We conclude that *Tsix *and pluripotency factors act synergistically to repress *Xist *in undifferentiated embryonic stem cells. Double mutants do not exhibit maximal levels of *Xist *expression, indicating that other pathways also play a role.

## Background

In female mammals a developmentally regulated process, X inactivation, ensures silencing of a single X chromosome, balancing levels of X-linked genes relative to males [1]. X inactivation is mediated by the cis-acting non-coding RNA Xist that is transcribed from and coats the inactive X chromosome (Xi) elect [2]. Coating by Xist RNA triggers epigenetic modifications that silence transcription and establish a heritable heterochromatic state [3].

X inactivation in the mouse occurs in two waves; imprinted X inactivation of the paternal X chromosome (Xp) that is initiated in two to four cell embryos and maintained in all cells until the blastocyst stage, and random X inactivation, initiated in the postimplantation epiblast. Embryo precursors in the inner cell mass (ICM) of the blastocyst reactivate Xp, reversing imprinted X inactivation and setting the ground state for the onset of random X inactivation [4,5]. XX embryonic stem (ES) cells, which are derived from the ICM, mirror this ground state, retaining two active X chromosomes [6,7]. In contrast extraembryonic trophectoderm and primitive endoderm lineages and cell lines derived thereof retain the imprinted X inactivation pattern through embryogenesis [8-11].

X chromosome reactivation also occurs in XX primordial germ cells during migration towards the genital ridges [12-14], and similarly during experimental reprogramming of XX somatic cells, either by cloning, cell fusion with pluripotent cells or induced pluripotent stem cell technology [15-17]. In all of these examples, including ICM cells, X reactivation is linked to extinction of Xist RNA expression from Xi. Xist-dependent reversibility of X inactivation is specific to pluripotent lineages and/or cell types as conditional knockout of *Xist *in somatic cells does not lead to X reactivation [18,19].

The mechanism underlying extinction of *Xist *expression in pluripotent cells is poorly understood. The antisense repressor *Tsix *is a candidate but deletion of the *Tsix *promoter in undifferentiated ES cells leads to only low levels of *Xist *upregulation and in a small proportion of cells [20,21]. Moreover *Tsix *expression is not observed in primordial germ cells (PGCs) at the time of X reactivation [13]. A second candidate is a Nanog/Oct4/Sox2 (NOS)-binding element located in *Xist *intron 1 [22]. Depletion of Nanog or Oct4 does indeed increase levels of Xist RNA. Set against this, a recent study found that deletion of the intron 1 NOS does not increase Xist RNA levels in undifferentiated XX ES cells, although there was an effect on X chromosome choice following differentiation *in vitro *[23]. In this study we have used a transgenic strategy to analyse the role of the intron 1 NOS and *Tsix *in repressing *Xist *in ES cells. We show that deletion of the intron 1 element moderately increases *Xist *expression in ES cells and that this effect is amplified by simultaneous deletion of *Tsix*. We conclude that *Tsix *and the intron 1 NOS function synergistically to repress *Xist *in undifferentiated ES cells.

## Results and Discussion

### Repositioning and inversion of the intron 1 NOS does not affect Xist regulation

**Figure 1:**
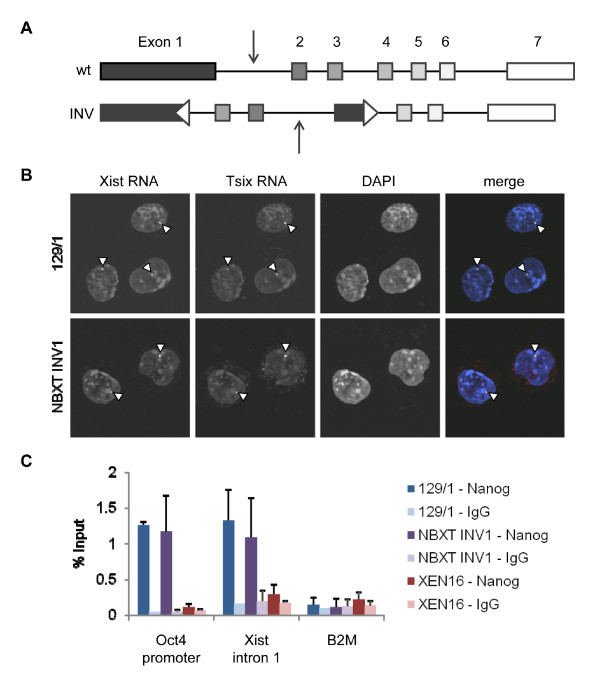
**Analysis of Nanog binding to *Xist *Intron 1 in ES cells carrying a targeted inversion in *Xist***. (A) Schematic representation of the wt and INV *Xist *alleles. Arrows indicate the position of the NOS binding site. (B) RNA FISH images showing *Xist *(green) and *Tsix *(red) expression in wt ES cells (129/1) and those carrying a targeted inversion in the *Xist *locus (NBXT INV1). Arrowheads point to punctate signal. DNA was counterstained with DAPI (blue). (C) Occupancy of the Oct4 promoter and *Xist *intron 1 by Nanog in wt ES cells (129/1) and NBXT INV1. XEN cells which do not express Nanog were used as a negative control. The promoter of the housekeeping gene *B2M *was used as a negative control for Nanog binding. DAPI: 4',6-diamidino-2-phenylindole; ES: embryonic stem; FISH: fluorescent *in situ *hybridisation; NBXT INV1: *Xist *allele carrying a targeted inversion; wt: wild type; XEN: extraembryonic endoderm.

A previous study demonstrated that acute downregulation of Oct4 in XY ES cells leads to rapid depletion of Oct4, Nanog and Sox2 proteins at the binding region of *Xist *intron 1 and considerable upregulation of *Xist *expression [22]. This effect, however, is observed only in 10% of cells and is accompanied by cell differentiation. To exclude the possibility of an indirect effect of Oct4/Nanog depletion on *Xist *regulation, we decided to directly test the role of NOS binding sites within *Xist *intron 1. For initial analysis we took advantage of a previously generated XY ES cell line (NBXT INV1) carrying a targeted inversion between exon 1 and intron 4 of the *Xist *locus [24]. In this cell line, the intron 1 element is retained but in a different position and in a reversed orientation (Figure 1A). We first analysed by RNA fluorescent *in situ *hybridisation (FISH) if *Xist *remained repressed in these cells. We found the culture to be indistinguishable from its parental wild-type counterpart (129/1), with one punctate signal in each cell (Figure 1B). As *Xist *remained repressed in these cells we then carried out chromatin immunoprecipitation (ChIP) to determine if *Xist *repression was maintained in the presence or absence of Nanog binding to intron 1 (Figure 1C). Again we found NBXT INV1 and 129/1 to be indistinguishable, with Nanog binding occurring at the Oct4 proximal promoter and *Xist *intron 1 to the same extent in both cell lines. As expected, Nanog binding was not detected in the extraembryonic endoderm (XEN) cell line where *Nanog *is not expressed ([8] and our unpublished data). Thus, it appears that reversing the orientation of the binding site in *Xist *intron 1 does not interfere with either Nanog binding or regulation of *Xist *expression.

### Deletion of Xist intron 1 within a P1 construct triggers Xist upregulation

Since inversion of *Xist *intron 1 did not disrupt its purported function we decided to delete the region by *Galactokinase (GalK*)-mediated recombineering [25] in a P1-derived artificial chromosome carrying the entire *Xist *genomic sequence plus 34.2 kb upstream of the Xist transcriptional start site (TSS) and 24 kb downstream of *Xist *exon 8, and therefore encompassing most of the known critical *Xist cis *regulatory elements. As a positive control we analysed ES cells transfected with the P1 construct with an inducible promoter (tetracycline responsive element; TRE) introduced at the *Xist *TSS. Addition of doxycycline caused a robust Xist transcription and accompanying chromosomal acquisition of histone modifications associated with the silenced state (Additional file 1).

**Figure 2:**
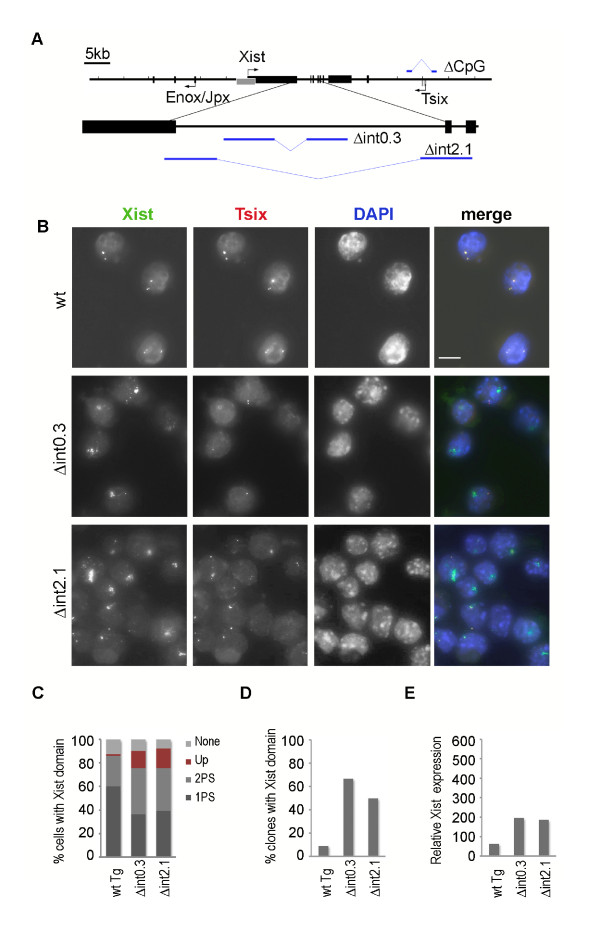
**Deletion of NOS binding region within *Xist *intron 1 causes moderate upregulation of transgenic *Xist *expression in undifferentiated XY ES cell lines**. **(A) **schematic representation of X inactivation centre region cloned into bacteriophage clone P1. *Xist *and *Tsix *exons are indicated as black and grey rectangles, respectively. The first three exons of *Enox/Jpx *are also shown. The direction of transcription for each locus is indicated by arrows. An enlarged region spanning *Xist *exons 1 to 3 is shown underneath the main schematic. Blue horizontal bars underneath indicate the position of homology arms used for recombineering. 0.3 kb (Δint0.3) and 2.1 kb (Δint2.1) sequences within intron 1 deleted from P1 clone by recombineering both encompass NOS-binding region. Blue lines above the main schematic indicate the position of the homology arms and the deleted region of the *Tsix *promoter (ΔCpG). **(B) **RNA FISH analysis of *Xist *and *Tsix *expression in undifferentiated XY ES clones carrying wt P1 construct (clone L5E2), P1 construct with small (Δint0.3, clone L9D7) or large deletion (Δint2.1, clone L7B2). Bar, 10 μm. **(C) **A graph showing proportional representation of four patterns of *Xist *expression in XY ES clones carrying P1 transgenes: light grey, no detectable *Xist *expression; red, upregulated Xist cloud; grey, two punctate Xist signals; dark grey, one punctate Xist signal. Average data for 12 clones of each genotype are shown. Individual clone data are shown in Figure 3A. **(D) **Graph showing a percentage of clones with upregulated *Xist*. **(E) **qRT-PCR analysis of *Xist *expression in XY ES clones carrying either wt P1 or P1 with 0.3 kb (Δint0.3) or 2.1 kb (Δint2.1) deletions in *Xist *intron 1. All data is normalised to β-actin transcript levels and presented relative to the wt XY ES (129/1) Xist RNA level. Average data for 12 clones of each genotype are shown. Individual clone data are shown in Figure 3B. ES: embryonic stem; FISH: fluorescent *in situ *hybridisation; NOS: Nanog/Oct4/Sox2; qRT-PCR: quantitative reverse transcription polymerase chain reaction; wt: wild type.

A bioinformatic search for NOS consensus sequences within the *Xist *locus revealed three potential Nanog-binding sites and one Oct4/Sox2-binding site located in close proximity to each other within intron 1 (data not shown). The identified sites lay within the region that showed the highest enrichment for Nanog and Oct4 proteins ([22] and our unpublished data). Based on this data we designed a strategy to remove the minimal region encompassing these sites in the P1 clone. The homology arms for recombineering were designed to delete 0.3 kb of the intron 1 region without introducing any foreign sequences (Figure 2A; see Methods). The resulting construct Δint0.3 as well as the parental P1 clone (wild type; wt) were co-lipofected with a selection plasmid carrying puromycin resistance under the control of mammalian *Phosphoglycerate kinase *promoter (pPGKpuro) into the 129/1 XY ES cell line and puromycin-resistant colonies were picked and analysed by PCR for the presence of a P1 construct. Twelve P1-positive clones for each construct were selected randomly for analysis of *Xist *expression.

**Figure 3:**
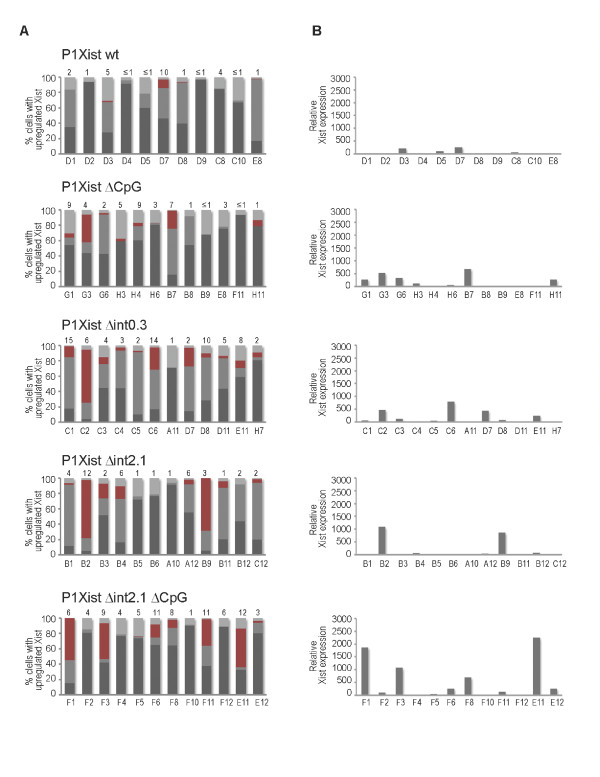
**Deletion of *Xist *intron 1 and the *Tsix *promoter causes upregulation of transgenic *Xist *expression in undifferentiated XY ES cell lines**. (A) Graphs showing proportional representation of four patterns of *Xist *expression in XY ES clones carrying P1 transgenes: light grey, no detectable *Xist *expression; red, upregulated Xist cloud; grey, two punctate Xist signals; dark grey, one punctate Xist signal. Each bar represents an individual clone. (B) qRT-PCR analysis of Xist expression in XY ES clones carrying P1 transgenes. All data is normalised to β-actin transcript levels and presented relative to the wt XY ES (129/1) Xist RNA level. Each bar represents an individual clone. ES: embryonic stem; qRT-PCR: quantitative reverse transcription polymerase chain reaction; wt: wild type.

RNA FISH analysis of Xist and Tsix transcripts showed an upregulated Xist domain is present in a proportion of cells in the majority of the clones carrying Δint0.3 (Figure 3A). The proportion of Xist domains varied considerably between different clones (0% to 69%) and the size and appearance of the domains varied between clones as well as between cells of the same clone (Figure 2A, 3A). Generally, domains were smaller than those observed in female somatic cells, but in some cells they were diffuse and occupied a large area of the nucleus. In contrast, all but one clone with the control wt P1 construct showed one or two punctate signals, corresponding to the endogenous and transgenic Xist (Figure 2B, 3A). The single exception, clone D6, showed a domain reminiscent of Xist in female somatic cells. However, an equivalent signal was detected with both Xist and Tsix probes (Additional file 2), suggesting that the P1 transgene integrated in multicopy in an open chromatin environment, leading to misexpression of both *Xist *and *Tsix *loci. This clone was therefore excluded from further analysis. Together, these results indicate that deletion of the NOS binding region leads to moderate upregulation of *Xist *expression in undifferentiated ES cells.

Our bioinformatic analysis revealed several other binding sites for Nanog, Oct4 and Sox2 spread throughout *Xist *intron 1. We decided to extend the deletion and remove 2.1 kb of intron 1 (Δint2.1) to test whether these other sites contribute to the repression of *Xist *(Figure 2A). RNA FISH analysis of a series of clones yielded results similar to those obtained for Δint0.3 (Figure 2B, C, Figure 3A). Once again, the degree of *Xist *upregulation varied between clones (0% to 76%) and the Xist domains observed were similar to those in Δint0.3 clones. This result indicated that the repressive function of intron 1 maps predominantly to the 0.3 kb minimal binding region.

We went on to analyse the degree of *Xist *upregulation by quantitative RT-PCR (qRT-PCR). As expected, clones that showed Xist domains by RNA FISH analysis also showed higher levels of *Xist *expression (Figure 3B). Further, our RT-PCR analysis of *Xist *exon-intron structure demonstrated that Xist RNA was spliced correctly (data not shown). On average, *Xist *was upregulated approximately four times over the level of Xist in P1 wt ES cells for both Δint0.3 and Δint2.1 (Figure 2E). Taking into account that only 50% to 60% of clones had demonstrated a substantial proportion of cells with an Xist domain (Figure 2D), the degree of *Xist *upregulation in clones with the domain was considerably higher (Figure 3B). Thus deletion of the binding region for Nanog, Oct4 and Sox2 located within the *Xist *intron 1 caused derepression of *Xist*, albeit to varying degrees between and within different clones.

### Simultaneous deletion of Xist intron 1 and the Tsix promoter facilitates derepression of Xist expression

The non-coding RNA Tsix is transcribed in an antisense orientation through the entire *Xist *locus and is regarded as a major repressor of *Xist *in undifferentiated ES cells [26]. However, a deletion of the *Tsix *promoter or premature termination of the Tsix transcript causes only limited *Xist *upregulation [20,21]. We hypothesised that *Tsix *and *Xist *intron 1 may function redundantly in repressing *Xist *in undifferentiated ES cells. To test this, a deletion of the *Tsix *promoter and the major transcriptional start site (ΔCpG) [26] was introduced by recombineering into the control P1 construct (ΔCpG) and in the P1 construct carrying the large intron 1 deletion (Δint2.1ΔCpG).

**Figure 4:**
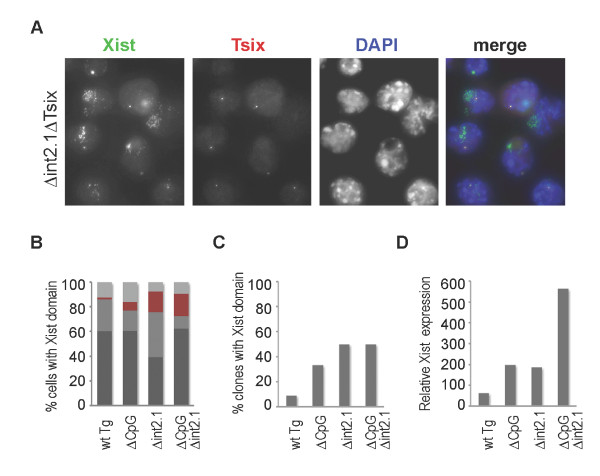
**Simultaneous deletion of the *Tsix *CpG island and *Xist *intron 1 further increases transgenic *Xist *activation in undifferentiated XY ES cell lines**. (A) RNA FISH analysis of *Xist *and *Tsix *expression in undifferentiated XY ES clones carrying P1 construct with deletion of the *Tsix *promoter (ΔCpG) and *Xist *intron 1 (Δint2.1, clone L8F1). (B) Graph showing proportional representation of four patterns of *Xist *expression in XY ES clones carrying P1 transgenes. Average data for 12 clones of each genotype are shown. Individual clone data are shown in Figure 3A. (C) A graph showing the percentage of clones with upregulated *Xist*. (D) qRT-PCR analysis of *Xist *expression in XY ES clones carrying either wt P1, P1 with deletion of *Tsix *promoter (ΔCpG), P1 with 2.1 kb (Δint2.1) deletion in *Xist *intron 1 or simultaneous deletion of *Tsix *promoter (ΔCpG) and *Xist *intron 1 (Δint2.1). All data is normalised to β-actin transcript levels and presented relative to the wt XY ES (129/1) Xist RNA level. Average data for 12 clones of each genotype are shown. Individual clone data are shown in Figure 3B. See Figure 2 for detailed annotation. ES: embryonic stem; FISH: fluorescent in situ hybridisation; qRT-PCR: quantitative reverse transcription polymerase chain reaction; wt: wild type.

Twelve clones carrying each P1 construct were analysed by RNA FISH for the presence of an Xist domain. Several ΔCpG clones had 1% to 10% of cells with a small Xist cluster, consistent with previous observations using *Tsix *mutant ES cells [21]. Two clones showed somewhat higher numbers of cells with a small Xist domain (23% and 36%), which was probably due to the site of integration or copy number (Figure 3A). Around half of the Δint2.1ΔCpG clones had an Xist accumulated domain, a result similar to Δint2.1 alone. However, the accumulated domain was generally larger in the clones that showed upregulation and the overall proportion of cells with the domain within those clones was higher (Figure 4A-C). qRT-PCR analysis confirmed the latter observation as average *Xist *expression was more than two-fold higher in Δint2.1ΔCpG compared with Δint2.1 or ΔCpG alone (Figure 3B, 4D).

While the results of these experiments clearly indicated that *Xist *intron 1 and *Tsix *contribute synergistically to the repression of *Xist *in undifferentiated ES cells, there was considerable variability of *Xist *derepression between different clones. A relatively high proportion of clones and/or cells carrying P1 with single or double deletions did not exhibit an Xist domain in spite of initial positive genotyping for the presence of a P1 transgene. There are several possible causes for this variability, namely copy number of the transgene, site of integration, orientation of transgenic copies and transgene instability. Using qPCR and Southern blot hybridisation we estimated copy number of *Xist *transgenes to vary between one and fourteen. There is a general correlation, in that clones with higher transgene copy numbers are more likely to show some degree of *Xist *upregulation. However, this is not absolute and some clones with just two copies of the transgene show much higher upregulation than clones with higher copy number.

**Figure 5:**
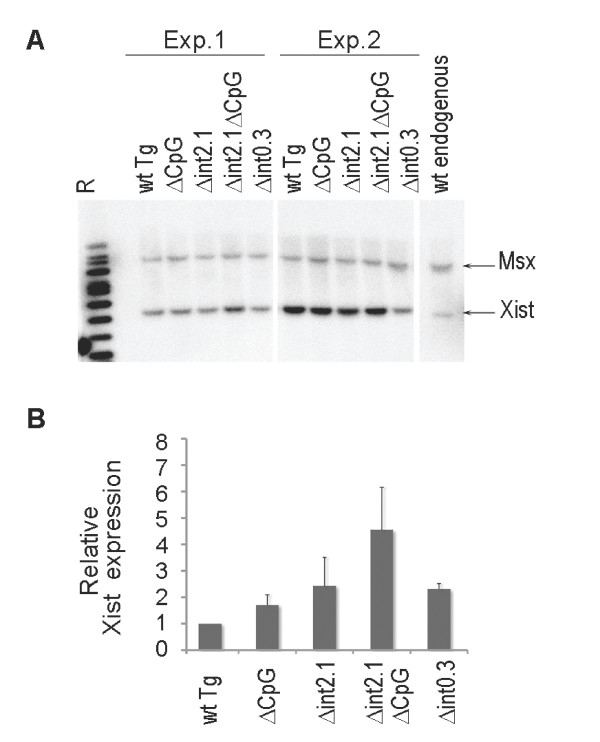
**Analysis of *Xist *expression in lipofected cell pools**. (A) Southern blot analysis of DNA extracted from the cell pools 12 days after lipofection of P1 transgenes into undifferentiated XY ES cells. Data for two experiments are shown. (B) qRT-PCR analysis of *Xist *expression in XY ES cell pools transfected with P1 transgenes. All data is normalised to β-actin and wt Xist transcript level in XY ES cells (129/1). The average value from three independent experiments (+ SEM) is shown for each transgenic genotype. ES: embryonic stem; Msx: homeobox, msh-like 1 autosomal gene used for internal normalisation; qRT-PCR: quantitative reverse transcription polymerase chain reaction; R: Raoul marker; wt: wild type.

Southern blot analysis revealed that the majority of clones have rearrangements, indicating transgene instability (Additional file 3). We reasoned that since the clonal analysis requires prolonged passaging of cells in culture, this could enhance frequency of transgene rearrangements due to selective pressure or/and transgene instability over time. To minimize this effect, we decided to use a different approach and analyse pooled clones after co-lipofection of P1 transgene with a pPGKpuro selective plasmid immediately after they have undergone a selection for transgene integration. This approach has the disadvantage that not every clone will contain a P1 construct, but assuming that all parameters are the same, pools with different P1 constructs will have similar lipofection efficiency and average transgene copy number. We performed the experiment on three different pools for each construct to account for experimental variability. Initially we analysed each pool individually by Southern blot hybridisation analysis to determine the average copy number and assess transgene integrity. As anticipated, pooled clones with minimal passaging time did not show any transgene rearrangements (Figure 5A). Transgene copy number varied between the experiments, but was broadly similar between the different pools within each experiment (Figure 5A).

RNA FISH analysis of the pooled clones confirmed the data from the analysis of individual clones, and pools carrying Δint2.1ΔCpG showed the highest proportion of cells with an Xist domain (not shown). To quantify this we analysed *Xist *upregulation in clone pools by qRT-PCR. Average data were obtained for three primer pairs along the Xist transcript (ex1, ex2-3 and ex4-5) for each of the pools. We observed variability in the degree of *Xist *upregulation between the experiments, however pools with Δint2.1ΔCpG consistently showed the highest *Xist *expression level (Figure 5B).

Taken together, our results suggest that the *Xist *intron 1 region is important for *Xist *repression in undifferentiated pluripotent cells, as proposed previously [22], but that it functions synergistically with *Tsix*, the two mechanisms acting redundantly. The latter finding may provide some explanation for the observations of Barakat and colleagues [23], who did not detect *Xist *upregulation in undifferentiated XX ES cells carrying deletion of the intron 1 NOS on one allele. Our data show that the intron 1 NOS behaves as a classical silencer element in that it can function in a distance and orientation independent manner.

Whilst our results provide clear evidence supporting a role of the intron 1 NOS in *Xist *repression, the *Xist *upregulation we observed from mutant transgenes is relatively variable, both between clones and within individual clones, and rarely occurs to the extent seen in XX somatic cells. It is possible that this variability and low expression is a consequence of selection against the cells which upregulate Xist and silence autosomal genes in *cis*, although arguing against this we did not observe increased lethality in clones with high transgenic Xist expression. A more plausible explanation is that other repressors and/or *Xist *activators play a role. Sado and colleagues [27] observed significant upregulation of the *Xist *promoter in ES cells carrying a deletion of a large region including much of *Xist *exon 1 and some of *Xist *intron 1. Whilst it is possible that this deletion disrupts normal function of the intron 1 NOS and/or *Tsix*, it is also possible that other unidentified functional sequences have been removed. Also relevant is that synergistic function of the intron 1 NOS and *Tsix *cannot account for *Xist *repression in PGCs as *Tsix *appears not to be expressed in this context [13,14], again suggesting that other factors can contribute. Finally, it is not known the degree to which levels of *Xist *activators, for example Rnf12 [23,28] or the Jpx/Enox ncRNA [29] could contribute to reduced *Xist *expression in pluripotent cells. A recent study indicates that pluripotency factors repress levels of Rnf12 [30], although set against this, ectopic expression of Rnf12 in ES cells with the intron 1 NOS deletion does not trigger *Xist *upregulation [23].

## Conclusion

Our results show that *Xist *repression in undifferentiated ES cells is controlled by synergistic and/or redundant mechanisms. Binding of pluripotency factors to the element in *Xist *intron 1 contribute to *Xist *repression, as does transcription of the antisense RNA, Tsix. However, ablation of these two pathways does not lead to complete derepression, indicating that other pathways must also be involved.

## Methods

### Cell culture

The 129/1 ES cell line [31] was grown as previously described [32]. ES cells were lipofected with P1 DNA using Lipofectamine 2000 (Invitrogen, UK) according to the manufacturer's instructions. The night before transfection, 1 × 10^6 ^cells were seeded in antibiotic-free medium on a well of a six-well plate. The cells were co-lipofected with 2 μg of P1 DNA and 50 ng of selective plasmid with the puromycin resistance gene under the PGK promoter with a 1:3 ratio between DNA and Lipofectamine 2000. The cells were trypsinised 24 h later and replated on a 90 mm Petri dish seeded with puromycin-resistant mitomycin (Sigma-Aldrich, UK)-inactivated feeder cells. Puromycin selection (2 μg/mL) was applied 48 h after lipofection. Puromycin-resistant colonies were either picked individually and expanded for analysis 12 days later or all colonies were pooled together and analysed 11 days after transfection.

The XEN16 cell line derived in house was cultured in Royal Park Memorial Institute (RPMI) 1640 medium supplemented with 10% FCS, 100 units/mL penicillin, 100 μg/mL streptomycin, 2 mM L-glutamine, 1 mM sodium pyruvate and 50 μM β-mercaptoethanol (all reagents from Invitrogen unless otherwise stated). This cell line was used as a negative control for ChIP with Nanog antibody (Cosmo Bio Co., Ltd, Japan) as XEN cells do not express *Nanog*.

### Recombineering

*GalK*-mediated recombineering in the P1 15503 (Incyte Genomics, USA) clone was performed essentially as previously described [25]. Arms of homology for each of the recombineering constructs were cloned into the pBluescript plasmid and the G*alK *gene was inserted in between. These *GalK*-carrying plasmids were used for the first round of recombineering to replace a region of interest with the *GalK *selective gene. pBluescipt plasmids with arms of homology only were used for the second round of recombineering to remove *GalK*. Primers used for PCR to amplify the arms of homology are listed in Additional file 4.

### RT-PCR analysis

RNA was isolated using TRIZOL reagent (Invitrogen) and treated with Turbo DNA-free reagent (Applied Biosystems, UK) according to the manufacturer's instructions. cDNA synthesis was primed from random hexamers (GE Healthcare, Life Sciences, UK) with Superscript III reverse transcriptase (Invitrogen). qRT-PCR was performed with SYBR Green PCR Master Mix (BioRad Laboratories, UK) on a Chromo4 Real-time PCR System (BioRad Laboratories). PCR primers and conditions were as described previously [33]. The data was normalised to β-actin and then to the 129/1 control ES cell line.

### RNA FISH analysis

RNA FISH was performed essentially as described previously [34]. p*Xist*, an 18 kb DNA fragment spanning the whole Xist transcript, was directly labelled using Spectrum Green-dUTP and nick translation kit (both from Abbott Diagnostics, Abbot UK). A Spectrum Red-dUTP (Abbott Diagnostics)-directly labelled 2.5 kb PCR fragment from the region immediately downstream from the ΔCpG deletion was used as a Tsix probe. Images were acquired on a Zeiss AX10 microscope equipped with AxioCam MRm charge-coupled device camera using AxioVision software (Carl Zeiss International, UK).

### Immunofluorescence

ES cells were trypsinised, rinsed with EC10 medium followed by a PBS wash and cytospun onto Superfrost Plus glass slides (VWR, UK) at 1800 rpm for 3 min (Cytospin centrifuge; Shandon, Pittsburgh). Immunofluorescence was then performed as described previously [35].

### Chromatin immunoprecipitation

Cells were trypsinised, washed with ice-cold PBS and fixed in 1% formaldehyde in PBS for 10 min at room temperature with constant rotation. The crosslinking reaction was quenched by the addition of 1/10 volume of 1.25 M glycine. After washing in ice-cold PBS, the cells were lysed in ChIP lysis buffer (50 mM Tris, pH 8.0; 10 mM EDTA; 1% SDS), containing protease inhibitors (Complete mini, Roche Diagnostic, UK). The lysates were sonicated using a Bioruptor sonicator (Diagenode, Belgium) to yield fragment sizes between 300 and 500 bp and stored at -80°C until immunoprecipitation was carried out. The lysate containing the chromatin was diluted 1:10 in dilution buffer (1% Triton X-100, 2 mM EDTA pH8.0, 150 mM NaCl, 20 mM Tris-HCl pH 8, protease inhibitors). 75 μg chromatin was then incubated with 4 μg antibody (anti-Nanog, Cosmo Bio; anti-Oct4 sc-8628X, Santa Cruz; immunoglobulin G, Abcam, UK or Sigma-Aldrich) overnight at 4°C and then with protein G agarose beads pre-blocked with salmon sperm DNA (Millipore (UK) Ltd) for 3 h at 4°C. The beads were washed four times in low salt wash buffer (0.1% SDS, 1% Triton X-100, 2 mM EDTA, 150 mM NaCl, 20 mM Tris-HCl pH 8, protease inhibitors) and once in high salt wash buffer (0.1% SDS, 1% Triton X-100, 2 mM EDTA, 500 mM NaCl, 20 mM Tris-HCl pH 8, protease inhibitors). Immunoprecipitated DNA was eluted from the beads by incubation in elution buffer (1% SDS, 0.1 M sodium bicarbonate) with 150 μg proteinase K and 50 μg RNaseA for 2 h at 37°C and overnight at 65°C. DNA was then isolated by standard phenol:chloroform extraction. qPCR analysis of isolated DNA was performed on Chromo4 Real-time PCR System (BioRad Laboratories) using primers and conditions listed in Additional File 4.

## Abbreviations

ChIP: chromatin immunoprecipitation; EDTA: ethylenediaminetetraacetic acid; ES: embryonic stem; FCS: foetal calf serum; FISH: fluorescent *in situ *hybridisation; *GalK: galactokinase; *ICM: inner cell mass; NOS: Nanog/Oct4/Sox2; PBS: phosphate buffered saline; PCR: polymerase chain reaction; PGC: primordial germ cell; PGK: phosphoglycerate kinase; qRT-PCR: quantitative reverse transcription PCR; RT: reverse transcription; TSS: transcriptional start site; wt: wild type; XEN: extraembryonic endoderm; Xist: X inactive specific transcript; Xi: inactive X chromosome; Xp: paternal X chromosome.

## Competing interests

The authors declare that they have no competing interests.

## Authors' contributions

TBN, CES and NB conceived of and designed the experiments. TBN, CES, JS, TAS and AT performed the experiments. TBN, CES, JS, TAS, AT, MH and NB analysed the data. MH contributed reagents and materials. TBN, CES and NB wrote the paper. All authors read and approved the final manuscript.

## Supplementary Material

Additional file 1 **An inducible P1 Xist transgene triggers repressive histone tail modifications upon induction with doxycycline**. (A) Schematic representation of XIC region cloned into bacteriophage clone P1 15503 (P1). Relative positions of *Xist *gene (blue rectangle), *Tsix *promoter and TSS (dark grey box and arrow) and TRE (red box and arrow) are shown. Arrows indicate the direction of transcription. (B) RNA FISH analysis of *Xist *expression (green) in an undifferentiated XY ES line carrying an inducible P1 *Xist *transgene before (-dox) and after 1 day (+dox) of treatment with doxycycline. (C) Representative examples of H3K27me3 and H2AK119u1 staining of an undifferentiated XY ES line carrying an inducible P1 *Xist *transgene after one day of treatment with doxycycline. Click here for file

Additional file 2 ***Xist *upregulation in the wt P1 clone D6 is caused by a different mechanism**. Representative examples of cells from P1 wt D6, P1 Δint2.1 B2 and P1 Δint2.1 ΔCpG F1 clones are shown. Note the presence of large upregulated Tsix domain co-localising with upregulated Xist domain in P1 wt D6 and absence of Tsix domain in the P1 deletion mutant clones. Green arrows point to the Xist domain and red arrows indicate the corresponding position the red channel (Tsix probe). Directly labelled full length Xist cDNA (Xist, Spectrum Green, Abbott Diagnostics) and 2.6 kb Tsix fragment non-overlapping with the ΔCpG deletion (Tsix, Spectrum Red, Abbott Diagnostics; 29.8 kb downstream from the *Xist *TSS) were used as probes. Click here for file

Additional file 3 **Analysis of *Xist *expression in P1 transgenic clones**. Representative examples of Southern blot analysis of genomic DNA extracted from the ES clones lipofected with P1 transgenes. Genotype of P1 clone used for lipofection is indicated above the blots. R, Raoul marker (MP Biomedicals UK); Msx, homeobox, msh-like 1 autosomal gene used for internal normalisation. Click here for file

Additional file 4 **Supplemental table 1**. File contains a list of primers and PCR conditions used for ChIP analysis and to amplify arms of homology in P1 recombineering assay. Click here for file
