# Supplementary figures and images for: Pluripotency factor binding and Tsix expression act synergistically to repress Xist in undifferentiated embryonic stem cells
Source: Epigenetics Chromatin. 2011 Oct 7;4:17. doi: 10.1186/1756-8935-4-17 (PMC3197471; doi:10.1186/1756-8935-4-17)

**A**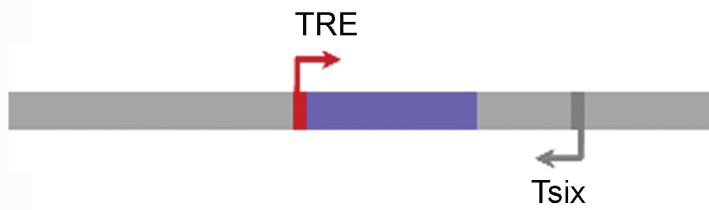**B**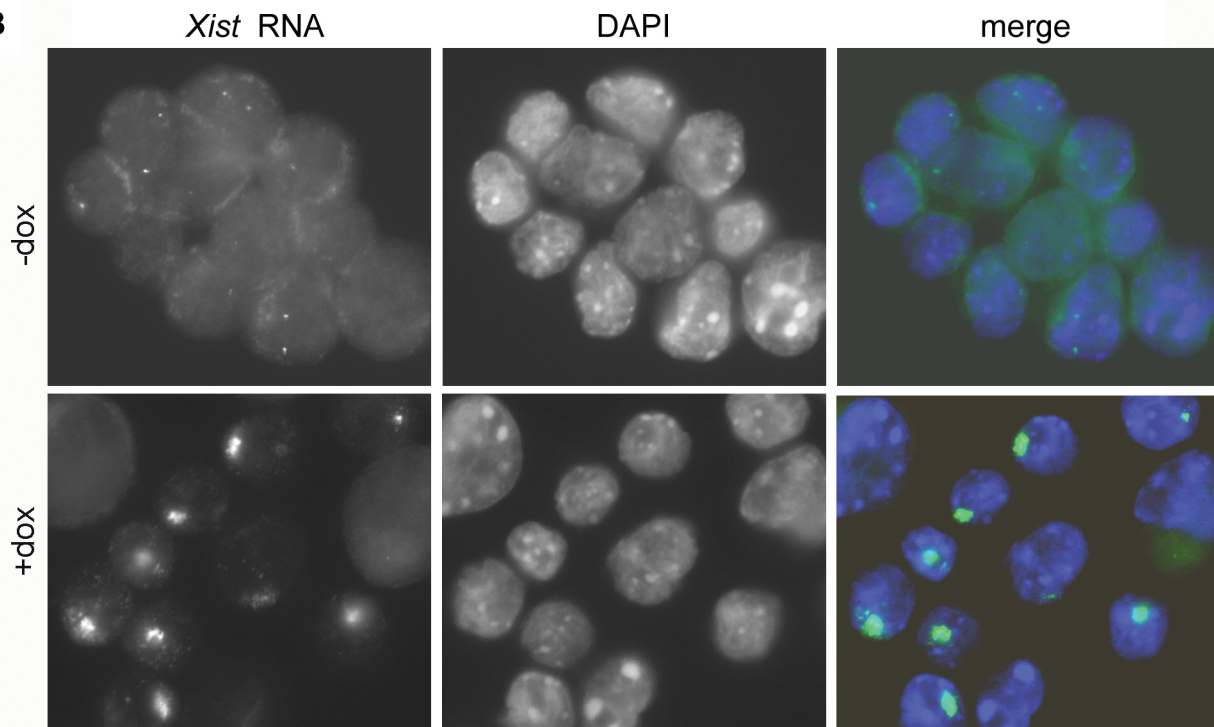**C**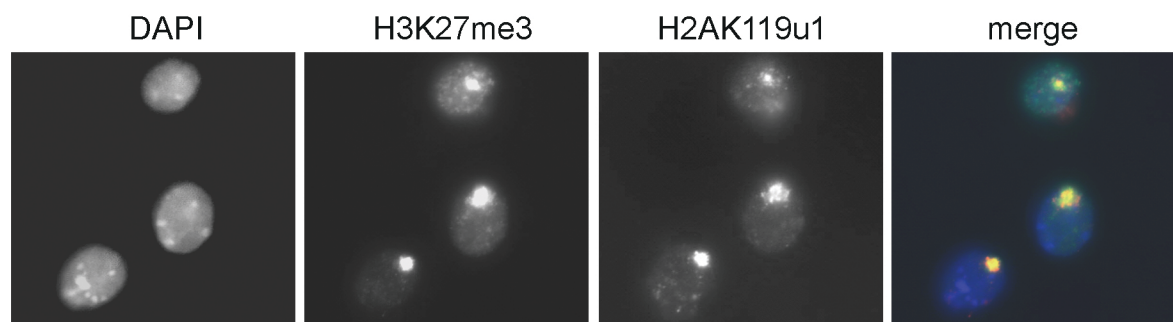

Supplement: Additional file 1 — An inducible P1 Xist transgene triggers repressive histone tail modifications upon induction with doxycycline. (A) Schematic representation of XIC region cloned into bacteriophage clone P1 15503 (P1). Relative positions of Xist gene (blue rectangle), Tsix promoter and TSS (dark grey box and arrow) and TRE (red box and arrow) are shown. Arrows indicate the direction of transcription. (B) RNA FISH analysis of Xist expression (green) in an undifferentiated XY ES line carrying an inducible P1 Xist transgene before (-dox) and after 1 day (+dox) of treatment with doxycycline. (C) Representative examples of H3K27me3 and H2AK119u1 staining of an undifferentiated XY ES line carrying an inducible P1 Xist transgene after one day of treatment with doxycycline. [file 1756-8935-4-17-S1.PDF]

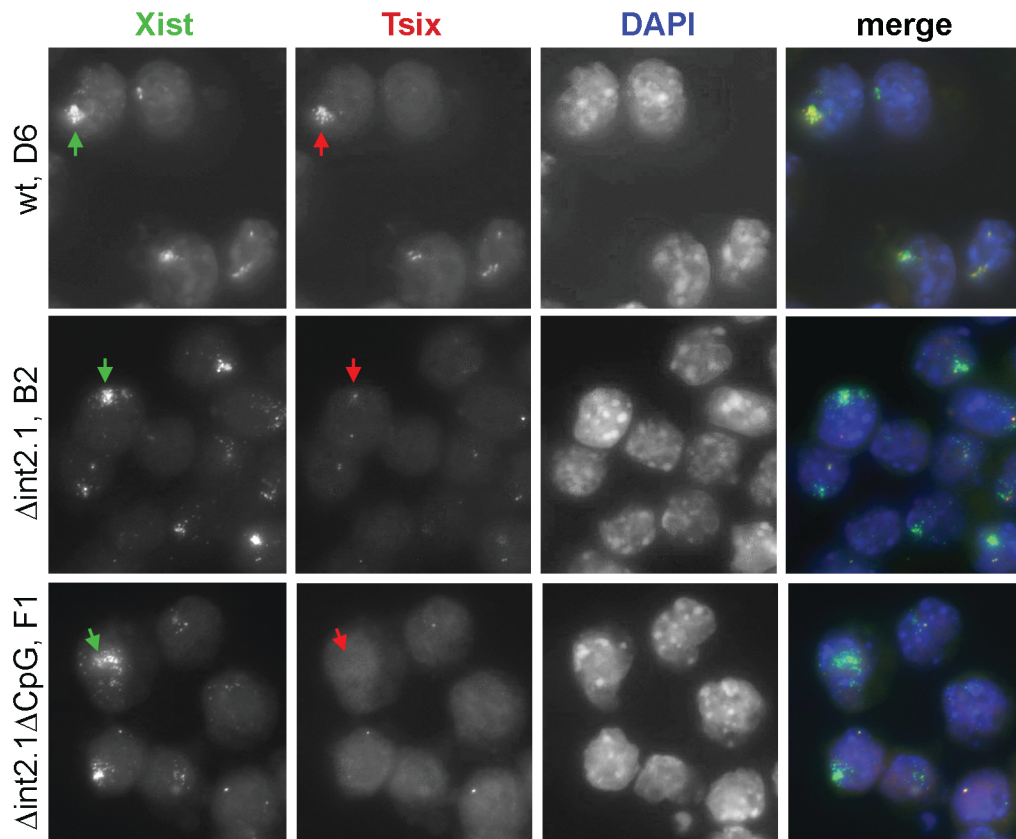

Supplement: Additional file 2 — Xist upregulation in the wt P1 clone D6 is caused by a different mechanism. Representative examples of cells from P1 wt D6, P1 Δint2.1 B2 and P1 Δint2.1 ΔCpG F1 clones are shown. Note the presence of large upregulated Tsix domain co-localising with upregulated Xist domain in P1 wt D6 and absence of Tsix domain in the P1 deletion mutant clones. Green arrows point to the Xist domain and red arrows indicate the corresponding position the red channel (Tsix probe). Directly labelled full length Xist cDNA (Xist, Spectrum Green, Abbott Diagnostics) and 2.6 kb Tsix fragment non-overlapping with the ΔCpG deletion (Tsix, Spectrum Red, Abbott Diagnostics; 29.8 kb downstream from the Xist TSS) were used as probes. [file 1756-8935-4-17-S2.PDF]

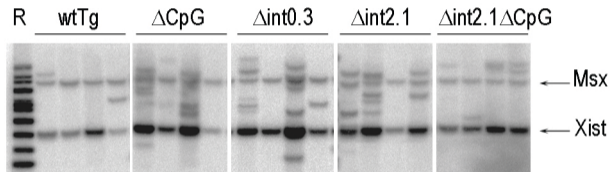

Supplement: Additional file 3 — Analysis of Xist expression in P1 transgenic clones. Representative examples of Southern blot analysis of genomic DNA extracted from the ES clones lipofected with P1 transgenes. Genotype of P1 clone used for lipofection is indicated above the blots. R, Raoul marker (MP Biomedicals UK); Msx, homeobox, msh-like 1 autosomal gene used for internal normalisation. [file 1756-8935-4-17-S3.PDF]
