# Supplementary material for: Pluripotency factor binding and Tsix expression act synergistically to repress Xist in undifferentiated embryonic stem cells
Source: Epigenetics Chromatin. 2011 Oct 7;4:17. doi: 10.1186/1756-8935-4-17 (PMC3197471; doi:10.1186/1756-8935-4-17)
Supplement: Additional file 4 — Supplemental table 1. File contains a list of primers and PCR conditions used for ChIP analysis and to amplify arms of homology in P1 recombineering assay. [file 1756-8935-4-17-S4.PDF]

Supplemental Table 1

Table S1. Primers and polymerase chain reaction (PCR) conditions

Gene	Forward Primer	Reverse Primer	Ta (^{0}C)
**ChIP primers**			
Xist, intron 1	ttgagatgctaaagaatgtgtt	gccaatggtgctaaggtgagac	60
Oct4 promoter	ggaactgggtgtggggaggttgta	agcagattaaggaagggctaggacgagag	60
B2M promoter:	acccgcctcacattgaaatcc	cgatcccagtagacggtcttg	60

Recombineering (arms of homology)

Tsix 5’ (575bp)	gagctctttggattaaagttgatgaa	gggcccgctttgcgagtttgaggttat	60
Tsix 3’ (555bp)	gagctctttcatgttcttcctt	gagctcttaaaagcaaagtatctcac	60
Δint0.3-5’ (528bp)	ttctttcaaccatttttactgc	gtgcctaggaagtactagagggtcatttg	60
Δint0.3-3’(427bp)	cctaggcacctagagctttcag	cctgaagatggtgatggcgagttg	60
Δint2.1-5’ (539bp)	ctcgaggaaaataatcatcacaacagc	ctcgagagcacacttccctcttta	60
Δint2.1-3’ (570bp)	gagctcgacgtctgactccctcttctg	gagctctgctggcagtccttgag	60
